# Isolation of Skin Leukocytes Uncovers Phagocyte Inflammatory Responses During Induction and Resolution of Cutaneous Inflammation in Fish

**DOI:** 10.3389/fimmu.2021.725063

**Published:** 2021-09-24

**Authors:** Amro M. Soliman, Taekwan Yoon, Jiahui Wang, James L. Stafford, Daniel R. Barreda

**Affiliations:** ^1^ Department of Biological Sciences, University of Alberta, Edmonton, AB, Canada; ^2^ Department of Agricultural, Food and Nutritional Science, University of Alberta, Edmonton, AB, Canada

**Keywords:** leukocytes, *Aeromonas*, inflammation, skin, immune response

## Abstract

Leukocytes offer a critical layer of protection to the host following skin infections. Delineating the kinetics of cutaneous leukocyte recruitment as well as their anti-microbial and regulatory profiles is challenging since it requires the isolation of adequate cell numbers and maintenance of their functional properties. Herein, we took advantage of a modified procedure to gain insights into the contributions of fish phagocytes through induction and resolution phases of acute cutaneous inflammation in goldfish (*Carassius auratus*). Our data shows early upregulation of pro-inflammatory cytokines and chemokines, which was paired with neutrophil-dominant leukocyte migration of neutrophils from circulation to the injury site. Recruited neutrophils were associated with high levels of reactive oxygen species (ROS). Following pathogen elimination, a reduction in ROS levels and pro-inflammatory cytokines expression preceded the resolution of inflammation. These results provide a better understanding of the cutaneous immune responses in fish. Moreover, the increased viability and functionality of isolated skin leukocytes opens the door to better understand a range of additional skin diseases.

## 1 Introduction

The skin is a primary site for interaction between an animal and its environment and is often the initial point of contact between the host and a number of pathogens. The unique structure and functions of the skin depend on the variety of its cell types, in addition to other hematopoietic cells derived from the circulation (1). Skin leukocytes are essential for clearing infection through complementary cellular and molecular responses (1). Initial pathogen exposure triggers an acute inflammatory response *via* binding of pattern recognition receptors (PRRs) expressed by tissue-resident cells to pathogen-associated molecular patterns (PAMPs) and damage-associated molecular patterns (DAMPs) (2, 3). Tissue-resident immune and connective tissue cells subsequently release pro-inflammatory cytokines as well as chemokines to recruit polymorphonuclear leukocytes (PMN), monocytes/macrophages and lymphocytes to the infection site from adjacent blood vessels (4, 5). The recruited cells combat invading pathogens through phagocytosis and anti-microbial mechanisms, e.g., nitric oxide (NO) and reactive oxygen species (ROS) (5). Following eradication of pathogens, a shift from pro-inflammatory to anti-inflammatory profile is essential for resolution of inflammation and activation of tissue repair machinery (5). Regulation of both pro-inflammatory and anti-inflammatory mediators is crucial for effective pathogen clearance along with minimal collateral tissue damage (6). Various mechanisms regulate the transition between induction and resolution of inflammation, including apoptosis of neutrophils and macrophage polarization towards an anti-inflammatory state (7, 8). During acute inflammation, macrophages internalize apoptotic neutrophils followed by downregulation of their pro-inflammatory profile, resulting in a reduction of infiltrating leukocytes and ROS levels (9–11). Subsequent to resolution of inflammation, upregulation of growth factors, cellular proliferation and activation of tissue repair pathways contribute to the restoration of tissue integrity and homeostasis (12).

*Aeromonas veronii* is a Gram-negative rod-shaped bacterium that was described for the first time in 1987 by Hickman-Brenner et al. (13). Despite being long-recognized as an important pathogen, it continued to impact several hosts, including fish and humans *via* inducing cutaneous infections associated with severe tissue damage, i.e., furunculosis (14–17). *A. veronii* is considered one of the most pathogenic species among *Aeromonas* spp., with *A. veronii biovar sobria* being the most pathogenic strain (18, 19). The bacterium has been isolated from several species of diseased fish (20–22). Infected fish are usually presented with well-characterized necrotic ulcers in addition to other signs of internal hemorrhage, abdominal distention and exophthalmia (21, 22). Though, not all of these signs are detected in infected fish, suggesting that pathogenicity depends on factors such as bacterial strain and fish species. *A. veronii* possesses several virulence factors that involve adhesins molecules, toxins, lytic enzymes, iron sequestering and quorum sensing systems (23–29).

Determining the functions and behaviour of leukocytes in connective tissue such as skin remains a challenge since it requires their isolation while considering the quantity and quality of the extracted cells. Protocols for leukocyte isolation from the skin of murine models (30) and fish (31–33) have been previously reported. A desirable cell extraction protocol should achieve high efficiency in cell harvesting concerning a high level of viability and functionality. Owing to its fibrous nature, enzymatic digestion of skin connective tissue is necessary to liberate cells. Several enzymes, including dispase, trypsin, and elastase were shown to modify the expression of surface receptors and functionalities of different cell types (34–36). Therefore, we used collagenase D enzyme, which was previously reported to have minor effects on surface markers and functions of isolated cells (37). Furthermore, we used MGFL-15 media specifically developed for *in vitro* cultivation of primary cells from carp and goldfish to maintain cell viability and maximize yield. With the help of the modified protocol, we managed to characterize skin leukocyte immune responses during induction and resolution phases of cutaneous inflammation in goldfish.

Our lab previously identified a period of neutrophilia in the peripheral circulation during the first 48 h subsequent to skin injury inoculated with *A. veronii* (20). This was followed by leukocyte infiltration into the injury area. However, limitations in our capacity to isolate and examine skin leukocytes prevented characterization of the kinetics of leukocyte recruitment into the skin and their anti-microbial and regulatory responses once at the injury site. A primary challenge was how to isolate sufficient numbers of leukocytes from the skin that remain viable and functional. Herein, we utilized a modified protocol that isolates high numbers of functional leukocytes with ~ 90% viability to characterize the recruitment kinetics of different leukocyte subsets in addition to their exerted anti-microbial responses during induction and resolution of cutaneous inflammation. Our data suggest that leukocytic infiltration, dominated by neutrophils, occurred at 24-48 hours post-infection (hpi), and was coupled to an increase in ROS production. Resolution of inflammation was evident by a reduction in both infiltrating leukocytes and ROS levels at 72 hpi. The results further provide insight into the cutaneous immune responses in fish skin.

## 2 Material and Methods

### 2.1 Ethics Statement

All animals were utilized according to the Canadian Council of Animal Care guidelines in addition to the University of Alberta Animal Care and Use Committee (Animal Use Protocol # 706). Goldfish were anesthetized using tricaine methanesulfonate (Aqualife TMS) at a concentration of 15-30 mg/L and pH of 7.4 to 7.6 and were euthanized by cervical dislocation.

### 2.2 Animals

Common goldfish (*Carassius auratus*), 10-12 cm in length, were purchased from Aquatic Imports in Calgary, Alberta. Fish were held in the Aquatic Facility of the Department of Biological Sciences, University of Alberta, on a simulated natural photoperiod (12 hours of light: 12 hours of dark). Fish were kept at 16°C in four continuous flow tanks (120 Liters in size) with 25 fish added to each tank. A number of five fish (n=5) were utilized per each time point. The water quality parameters throughout the experiment were maintained as follows; pH at 7.2–8.0 and dissolved oxygen at 5.5–6.5 PPM. Fish were fed once daily with 1.5 mm floating feed pellets.

### 2.3 Bacteria

*Aeromonas veronii* was previously isolated and identified by our lab from natural lesions found on goldfish held in the Aquatics facility (20). For the preparation of bacterial culture, frozen bacteria were inoculated into a 5mL of sterile trypticase soy media (BD Biosciences) and cultured overnight at room temperature in a tube shaker.

### 2.4 Wound Creation and Bacterial Inoculation

Following the anesthetizing of fish, scales on the mid-line of the left side of the fish were removed, and a 5x5 mm scratch wound was made with sterile fine-grit sandpaper. The wound was inoculated with 10 µL of *A. veronii* culture broth (concentration of 4.1 x 10^8^ CFU/mL) prior to returning the fish to water. Fish were held in flow-through 16°C water for 72 - 96 h, depending on the experiment. At indicated time points, fish were anesthetized and euthanized for wound tissue collection.

### 2.5 Histopathological Analysis

Tissue surrounding the wound area (2x2 cm) was collected and placed in 10% neutral-buffered formalin (Sigma) for 24 h. Following fixation, tissues were placed in 75% ethanol at 4°C. Tissues were subsequently processed overnight in a series of ethanol, toluene, and wax washes using a Leica TP1020 benchtop tissue processor. Paraffin-embedded tissues were left to harden and sectioned using a microtome.

Before staining, all the slides were deparaffinized using two rounds of toluene (5 min each). Slides were then washed using subsequent rounds of 100%, 90%, 70%, and 50% ethanol, and water for 2 min each. For Hematoxylin & Eosin (H&E) stain, slides were put in Haematoxylin solution for 20 min and washed in tap water for 5 min. Slides were stained in Eosin solution for 5 min and washed for 5 min in tap water. For Masson’s Trichrome stain, slides were stained with Lugol’s iodine for 5 min, decolorized with 5% sodium thiosulphate, and washed with tap water until they were clear. Slides were then stained with Wiegert’s iron hematoxylin for 20 min, decolorized with 1% acid alcohol and tap water, stained with 1% ponceau-fuchsin for 5 min, mordant in 1% phosphomolybdic acid for 5 min, then differentiated with 1% acetic acid. Finally, for both H&E and Masson’s Trichrome, slides were dehydrated in series of alcohol, cleared in xylene, mounted with DPX. Images were taken on a Leica DM1000 confocal microscope.

### 2.6 Isolation of Leukocytes From Fish Skin

Protocols for leukocyte isolation from fish skin (31–33) were previously reported. Herein, we present a modified protocol that utilizes enzymatic digestion and gradient centrifugation to extract immune cells from fish skin. In this protocol, we used MGFL-15 media specifically developed for *in vitro* cultivation of primary cells from carp and goldfish (**Table 1**), in addition to collagenase D for tissue digestion to maintain cell viability and maximize yield. The protocol is as follows:

**Table 1:** Components of MGFL-15 media.

Component	Quantity
KH2PO4	0.69 g
K2HPO4	0.57 g
NaOH	0.75 g
NaHCO3	0.34 g
HEPES	7.00 g
L-glutamine	0.584 g
Bovine Insulin	0.01 g
L-15 media	1L
10x Hank’s Balanced Salt Solution	80 mL
Nucleic acid precursor solution	20 mL
MEM amino acid solution	25 mL
MEM non-essential amino acid solution	25 mL
Sodium pyruvate solution	25 mL
MEM vitamin solution	20 mL
β-mercapto-ethanol	7 μL
MilliQ water	fill to 2L

Wound area was dissected and skin tissue was added into a petri dish containing cold sterile 1 x PBS^-/-^ (no calcium/no magnesium). Using sterile scissors, skin was cut into small pieces (~2mm^2^) and washed with cold 1 x PBS^-/-^ to avoid blood contamination. Skin pieces were moved into a 50 mL tube containing 10 mL of MGFL-15 media and the tube was added to a shaker for 30 minutes at room temperature. Content of the tube was strained through a sterile 70 µm cell strainer (Sigma). Then, skin pieces were collected and added into a new 50 mL tube with 10 mL of complete MGFL-15 media (with 5% (vol/vol) fetal bovine serum (FBS), 100 U/mL penicillin, and 100 μg/mL streptomycin) containing collagenase D (Sigma) (0.18 mg/mL). Then, the tube was added to a shaker for 120 minutes at room temperature. Content of the tube was strained through a sterile 70 µm cell strainer and flow-through containing cells was collected and washed with MGFL-15 media. Collected cell suspension was layered into a 51/34% discontinuous percoll density gradient (GE Healthcare) and centrifuged at 400 x g for 25 minutes at 4°C. Using an electronic pipette, slowly discard the upper layer and carefully collect the interface into a new 15 mL tube. Cells were washed twice with MGFL-15 media to be ready for downstream analysis.

### 2.7 Cell Staining

A 100 μL volume of cell suspension was added into a spin column and centrifuged onto a glass slide at 40 x g for 6 min using a Cytospin 4 cytocentrifuge (Shandon, ThermoFisher). For Hema 3 and Sudan Black staining, cells were stained according to the manufacturer’s specifications (Fisher HealthCar -Hema3 Fixative and Solutions and Sudan Black B, Sigma, respectively). Slides were visualized at 1000x magnification (with oil immersion) on a Leica DM1000 confocal microscope. Cellular subpopulations were counted based on the cellular morphology and Sudan Black staining. At least 200 cells were counted per sample.

### 2.8 Viability Assay

**Figure 1:**
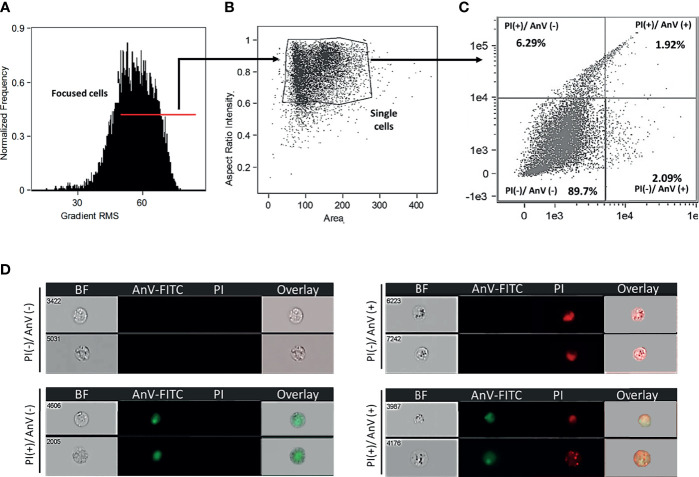
Goldfish leukocytes isolated from *A. veronii* skin injury site showed 90% viability. **(A)** Cells evaluated in an ImageStream Mk II Imaging Flow Cytometer (Amnis) were gated using gradient RMS to identify focused cells. Single cells **(B)** were subsequently evaluated for cell death **(C)** based on propidium iodide (PI) and annexin V (AnxV FITC) staining. **(D)** Representative images show healthy cells [PI(-)/AnxV(-)], as well as those undergoing apoptosis or necrosis [PI(+)/AnxV(-)], [PI(-)/AnxV(+)], [PI(+)/AnxV(+)]. BF, bright field.

Leukocytes were added to a 5mL round bottom tube (BD Falcon) at a density of 5x10^5^ cells and centrifuged at 350 x g for 8 min at 4°C. Cells were washed twice with 1x Annexin V Binding Buffer (BD Biosciences, Cat# 556454), resuspended in 200 μL 1x Annexin V Binding Buffer, and incubated for 30 min in the dark with 5 μL FITC Annexin V (BD Biosciences, Cat# 560931) and 4 μL propidium iodide (Sigma, Cat# P4864) diluted 1:10 in 1x Annexin V binding buffer. Finally, leukocytes were washed with 500 μL of 1x Annexin V Binding Buffer and fixed with 1% formaldehyde. Prior to analysis, leukocytes were washed twice with 1 x PBS^-^/^-^, centrifuged at 350 x g for 5 min at 4°C, and the supernatant was decanted. Data was acquired using ImageStream Mk II Imaging Flow Cytometer (Amnis) and analyzed using IDEAS Image Data Exploration and Analysis Software (Amnis). A minimum of 1x10^4^ events was acquired. Leukocytes were gated based on the normalized frequency of a fluorescent minus one sample. The reported protocol isolates skin leukocytes with ~ 90% viability (**Figure 1**).

### 2.9 ROS and NO Functional Assays

Cells were added to a 5 mL round bottom tube (BD Falcon) at a density of 5x10^5^ cells and centrifuged at 350 x g for 8 minutes at 4°C, then resuspended carefully into 500 μL MGFL-15 media. Cells were incubated in the dark for 30 min with 0.5 μL of CellROX™ Deep Red Reagent (Thermo Fischer, Cat# C10491), 1 μM DAF-FM (4-Amino-5-Methylamino-2’,7’-Difluorofluorescein) (Thermo Fischer, Cat# D23844), and 4 μL of propidium iodide (Sigma. Cat# P4864) diluted 1:10 in MGFL-15 media. Cells were washed 2 times with 1 x PBS^-/-^, and then fixed with formaldehyde 1%. After that, cells were centrifuged at 350 x g for 5 min at 4°C, and supernatant was removed. Data was acquired using ImageStream Mk II Imaging Flow Cytometer (Amnis) and analyzed using IDEAS Image Data Exploration and Analysis Software (Amnis). A minimum of 1x10^4^ events was acquired. Cells were gated based on the normalized frequency of a fluorescent minus one sample.

### 2.10 Gene Expression Analysis

#### 2.10.1 RNA Extraction

Goldfish were anesthetized and euthanized prior to collecting wound tissue. Tissues were homogenized in 1 mL of Trizol Reagent (Invitrogen, ThermoFisher) using a PRO 200 double insulated blade disruption homogenizer. One mL of each homogenized sample was transferred to respective microfuge tubes along with 100 μL of 1-bromo-3-chloropropane (Sigma). Samples were vortexed and kept on ice for 5 min, and centrifuged at 12,000 x g for 15 min at 4°C. The aqueous layer was collected in a new labeled tube. 100 μL of isopropanol was added to each tube and mixed by inversion before being stored at -80°C overnight. Tubes were centrifuged at 12,000 x g at 4°C for 10 min, supernatant removed, and RNA pellet was washed with 75% ethanol. After centrifugation at 7,500 x g for 5 min, the supernatant was discarded. The pellet was left to dry for 5 to 10 min. Samples were then resuspended in 30 μL of nuclease-free water (Ambion). cDNA was synthesized using iScript™ cDNA Synthesis Kit (Biorad). Then, cDNA samples were either used immediately or stored at -20°C for qPCR analysis.

#### 2.10.2 Quantitative (q) PCR Conditions

qPCR was performed using QuantStudio 6 Flex Real-Time PCR System (Applied Biosystems). In a 10 µL reaction mix, 5 µL SYBR green reagent mix, 0.5 µL of both forward and reverse primers (final concentration is 0.5 µM) and 2.5 µL of cDNA were added. cDNA was then analyzed by quantitative PCR. β-actin was used as an endogenous control. Relative quantification (RQ) analysis was performed. RQ values were normalized against gene expression on day 0. Primers used in qPCR are listed in **Table 2**.

**Table 2:** Primer sequences and accession numbers for q-PCR.

PRIMER	SEQUENCE (5’-3’)	ACCESSION NUMBER
*BACTIN* Forward	GAC CAA CCC AAA CCT CTC AA	AB039726
*BACTIN* Reverse	AGT CAA TGC GCC AAA CAG A	
*IL10* Forward	CAA GGA GCT CCG TTC TGC AT	HQ259106
*IL10* Reverse	TCG AGT AAT GGT GCC AAG TCA TCA	
*TNFA*2 Forward	TCA TTC CTT ACG ACG GCA TTT	EU069817
*TNFA*2 Reverse	CAG TCA CGT CAG CCT TGC AG	
*IL1B2* Forward	GAT GCG CTG CTC AGC TTC T	KC771268
*IL1B2* Reverse	AGT GGG TGC TAC ATT AAC CAT ACG	
*HSP27* Forward	GAT TCC ACC AGA CAT CGC CA	DQ872651
*HSP27* Reverse	ATT CCC AAC TCC ACC ATG TG	
*HSP70* Forward	GCT GGC TGA CAA AGA GGA GT	AB092839
*HSP70* Reverse	TGG CAT CCC TCC CTG ATA CA	
*TGFB* Forward	GTA CAC TAC GGC GGA GGA TTG	EU086521
*TGFB* Reverse	CGC TTC GAT TCG CTT TCT CT	
*INOSA* Forward	TTG GTA CAT GGG CAC TGA GAT T	AY904362
*INOSA* Reverse	CCA ACC CGC TCA AGA ACA TT	
*VEGF* Forward	ATG AGA ACC ACA CAG GAC GGG ATG TA	XM026228403
*VEGF* Reverse	CGA GAG CTG CTG GTA GAC ATC ATT	
*CXCL8* Forward	CTG AGA CTT TAC AGT GTG AGT GTG AGT TTG GAA	HM355573
*CXCL8* Reverse	TGG TGT CTT TAC AGT GTG AGT TTG G	

### 2.11 Statistical Analysis

Statistics were performed using non-parametric Kruskal–Wallis test and Dunn’s test for multiple comparison in Prism 7 software (GraphPad Prism).

## 3 Results

### 3.1 An Acute Inflammatory Response Characterized by Upregulation of Pro-Inflammatory Mediators and Neutrophil-Centric Leukocyte Recruitment Was Detected Following Cutaneous Injury

**Figure 2:**
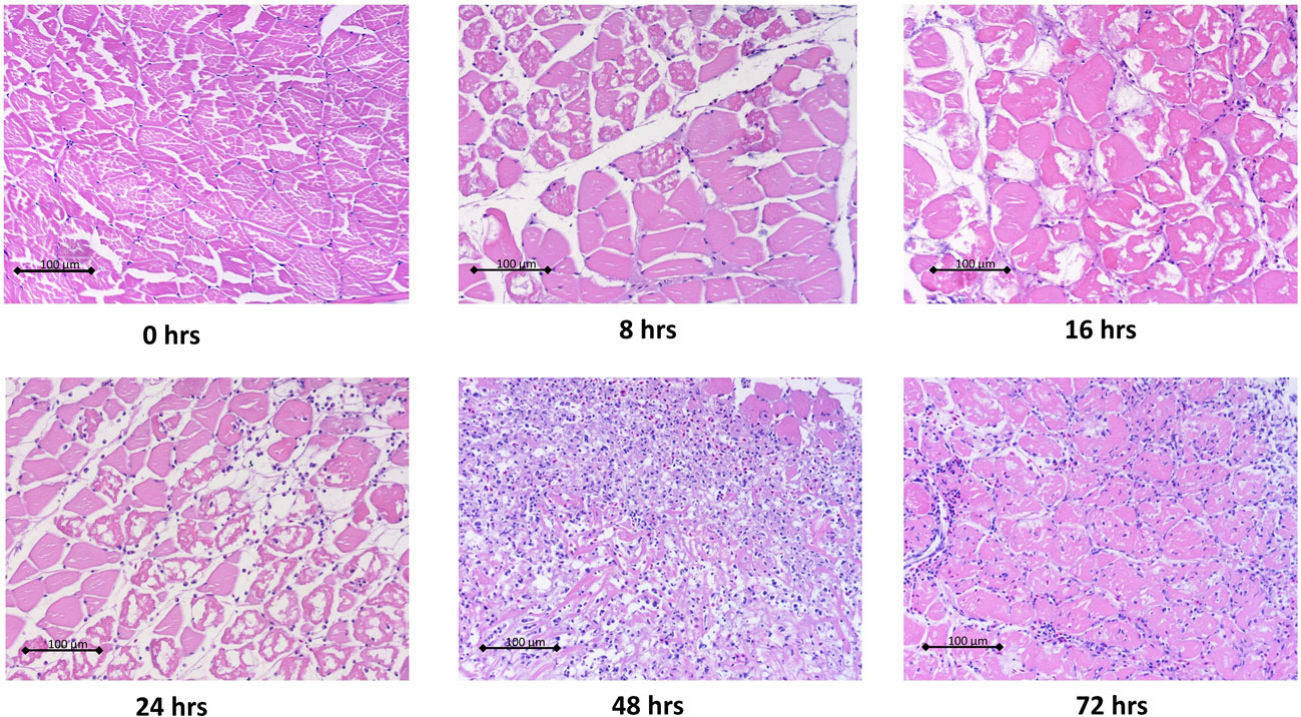
Histopathological analysis of wound tissue showed gradual recruitment of leukocytes to cutaneous infection site. Goldfish were wounded on the skin and inoculated with *A. veronii*. Wound tissues were collected at indicated time points. Tissues were fixed in 10% formalin then sectioned, stained and imaged. Representative hematoxylin and eosin stained sections are shown at 20x magnification.

**Figure 3:**
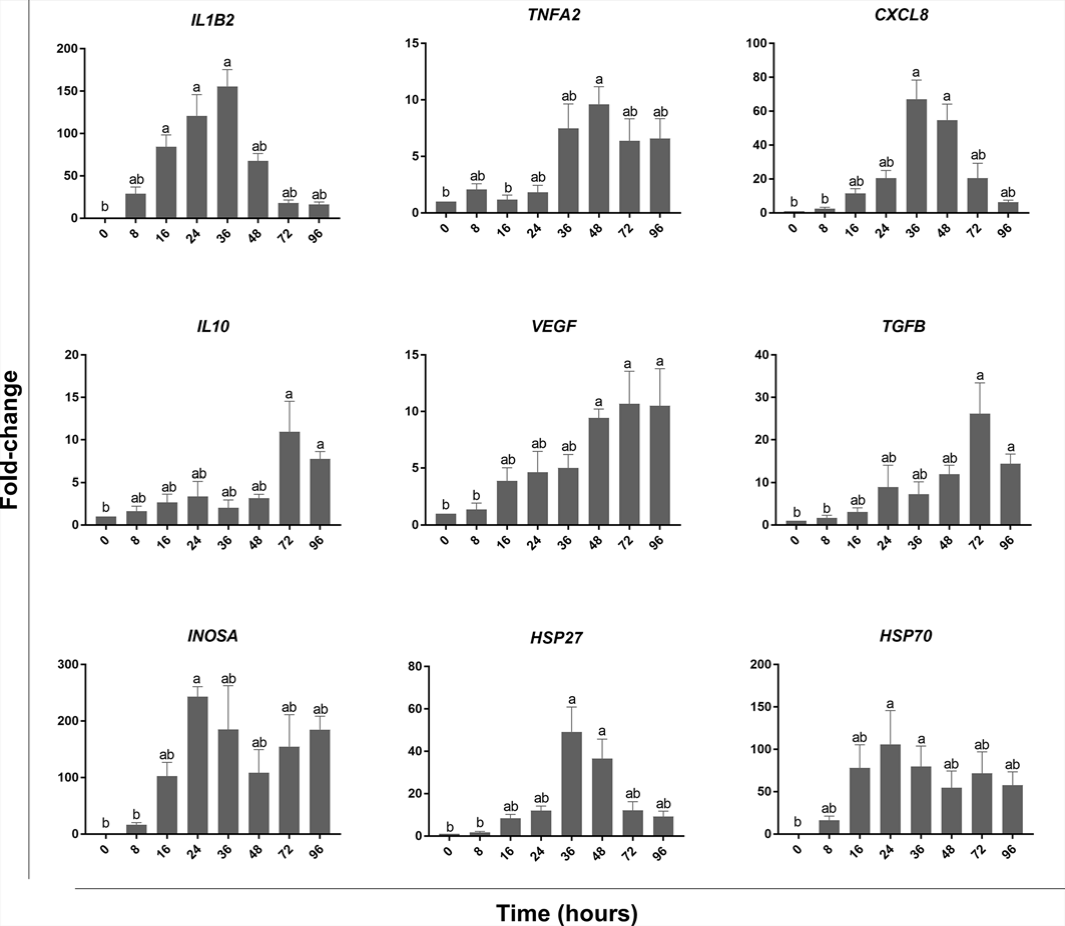
qPCR analysis of wound tissue revealed gene expression kinetics of classical pro-inflammatory and pro-resolution mediators. At each of the indicated time points, wound tissue was collected, RNA extracted, and cDNA made. qPCR was used to evaluate the expression levels of pro-inflammatory cytokines: *TNFA* & *IL1B*, anti-inflammatory cytokines: *TGFB* & *IL10*, chemokines: *CXCL8*, growth factors: *VEGF*, heat shock proteins: *HSP27* & *HSP70*, and *INOSA*. All statistical results correspond to a significance level of *P<0.05* using Kruskal–Wallis test followed by the Dunn’s test for multiple comparison. Graph bars represent the mean with error bars representing SEM. Different letters indicate statistical differences between groups; n=5.

**Figure 4:**
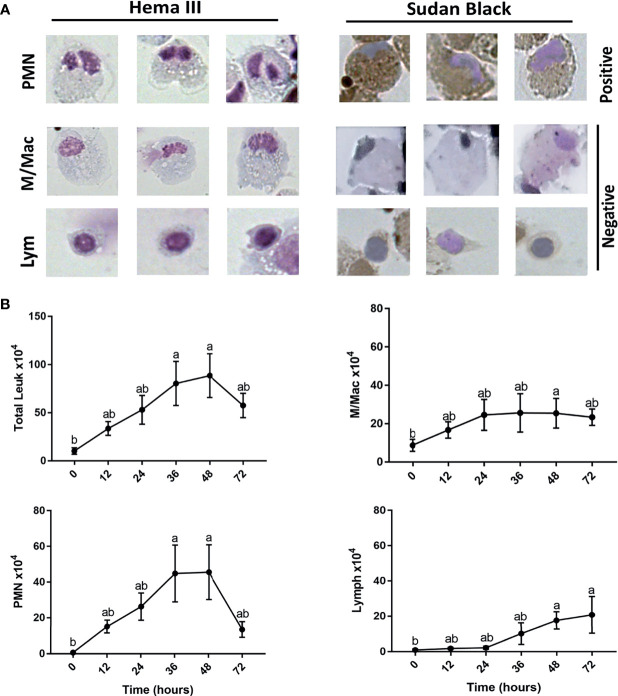
Kinetics for neutrophil, monocyte/macrophage, and lymphocyte subsets recruitment to cutaneous injury site. **(A)** Representative images show leukocytes stained with both Hema3 and Sudan Black stains. PMN (polymorph nuclear leukocytes)/Neutrophils are positive for Sudan Black staining, while monocytes/macrophages (M/Mac) and lymphocytes (Lym) are Sudan Black negative. **(B)** Total number of leukocytes, PMN, macrophages/monocytes, and lymphocytes isolated from wound tissue. At each indicated time point, leukocytes were isolated and counted using a hemocytometer. Then, cells were fixed on slides using Cytospin and stained with Sudan Black stain. At least 200 cells were counted to determine the proportion of individual leukocyte subsets, which would then be used to determine total cell numbers. All statistical results correspond to a significance level of *P<0.05* using Kruskal–Wallis test followed by the Dunn’s test for multiple comparison. Points represent the mean with error bars representing SEM. Different letters indicate statistical differences between groups; n=5.

Histopathological analysis showed that leukocytes were recruited to the injury site gradually to reach a peak at 48 hpi (**Figure 2**). Cellular recruitment correlated with upregulation of gene expression of classical pro-inflammatory cytokines and chemokines. For example, interleukin 1 beta (*IL1B*) was markedly upregulated at 16 – 36 hpi while tumor necrosis factor alpha (*TNFA*) showed a later upregulation at 36 and 48 hpi (**Figure 3**). To gain added resolution into the kinetics of infiltration of different leukocyte subpopulations, we isolated skin leukocytes from wound tissue. Consistent with our histopathological analyses above, the number of total leukocytes isolated from the skin infection site gradually increased to reach maximum levels between 36 to 48 hpi (**Figure 4B**). Among recruited cells, neutrophils, as expected, accounted for the majority of infiltrating leukocytes (> 56%) compared to monocytes/macrophages (~ 29.5%) during the first 48 hpi (**Figure 4B**). Limited numbers of neutrophils existed at the infection site at 0 hpi; though, they increased significantly to ~ 29 x 10^4^ at 24 hpi and then to ~ 45 x 10^4^ at 36 and 48 hpi, making neutrophils the main cells to respond and to be recruited to the infection site. Characterization of gene expression profiles at the wound site showed early upregulation of neutrophil chemoattractant released by tissue-resident cells, C-X-C Motif Chemokine Ligand 8 (*CXCL8*)/*IL8* 36-48 hpi (**Figure 3**), correlating well with neutrophils recruitment.

Other leukocytes such as monocytes/macrophages increased at the wound site at a relatively lower rate than PMN. For instance, we observed an increase in the number of monocytes/macrophages by ~ 17 x 10^4^, while there was a marked rise in neutrophil number by ~ 44 x 10^4^ at 36 hpi compared with the basal levels (**Figure 4B**). Interestingly, monocytes/macrophages were the dominant population of leukocytes following the resolution of inflammation at 72 hpi (**Figure 4B**). Macrophages also dominated the leukocyte population residing in the skin tissue with limited numbers of both neutrophils and lymphocytes at 0 hpi (**Figure 4B**). Lastly, lymphocytes infiltrated the wound site gradually to reach a significant number at 48 and 72 hpi (**Figure 4B**).

### 3.2 Activation of Anti-Inflammatory Program Was Associated With a Neutrophil-Dominated Decline in Skin Leukocytes and Resolution of Inflammation to be Followed by Tissue Repair

Resolution of inflammation is critical to prevent chronic inflammatory conditions and to initiate an effective tissue repair. Control of inflammation is demonstrated by downregulation of pro-inflammatory cytokines and leukocyte recruitment. Histopathological analyses and cell counts showed a reduction in the number of leukocytes in the wound area at 72 hpi (**Figures 2**, **4B**), which was driven by a sharp decline in neutrophils (**Figure 4B**). We further observed a substantial reduction in the gene expression of pro-inflammatory cytokines, e.g., *TNFA* and *IL1B* and chemokines (*CXCL8*) at 72 and 96 hpi (**Figure 3**). This was associated with a remarkable upregulation of crucial anti-inflammatory cytokines such as transforming growth factor (*TGFB*) and *IL10* (**Figure 3**). *TGFB* and *IL10* mediate robust pro-resolution functions by suppressing pro-inflammatory cytokines and chemokines expression, resulting in induction of pro-resolution events (38–40).

Although we detected a significant decline in neutrophils, macrophages/monocytes and lymphocytes were the dominant populations of leukocytes residing the wound tissue at 72 hpi (~ 40% and ~ 35%, respectively). Lymphocytes are involved in the induction of adaptive immune responses along with various functions related to tissue repair (41, 42). Meanwhile, macrophages secrete pro-resolution cytokines and growth factors, including vascular endothelial growth factor (*VEGF*) to control inflammation and promote wound healing (43). Our data showed a significant increase in the expression of *VEGF* at 48, 72 and 96 hpi. *VEGF* is crucial for enhancing vascular permeability, chemotaxis, reepithelialization, collagen deposition and angiogenesis (44). In addition to growth factors, macrophages and other tissue-resident cells produce heat shock proteins (HSP) to protect tissue against stress-induced misfolded proteins (45). We observed a significant upregulation of *HSP27* at 36 and 48 hpi; meanwhile, *HSP70 was upregulated at 24 and 36 hpi* (**Figure 3**).

### 3.3 Recruited Leukocytes Exert Differential Anti-Microbial Responses During Induction and Resolution of Inflammation Characterized by Dramatic Changes in ROS Levels

**Figure 5:**
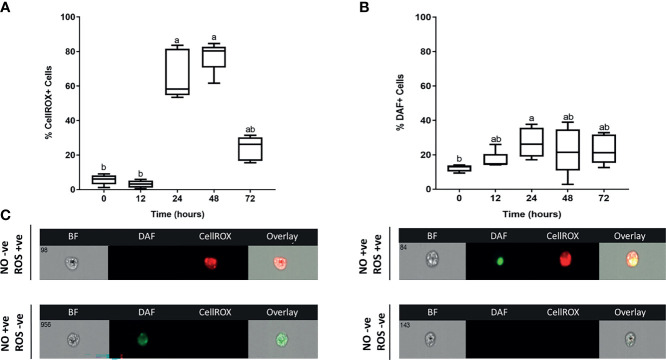
Kinetics of ROS and NO antimicrobial responses exerted by skin leukocytes during induction and resolution phases of cutaneous inflammation. Percentage of leukocytes associated with **(A)** reactive oxygen species (ROS); **(B)** nitric oxide (NO). At indicated time points, leukocytes were isolated from goldfish skin, then incubated with DAF-FM (detects NO), CellROX (detects ROS) and propidium iodide (PI) for 30 minutes. Using image flow cytometry, intensity of DAF-FM and CellROX was detected. All statistical results correspond to a significance level of *P<0.05* using Kruskal–Wallis test followed by the Dunn’s test for multiple comparison. A boxplot showing spread of data with their median. Different letters indicate statistical differences between groups; n=5. **(C)** Representative images from ImageStream MKII flow cytometer denote positive or negative DAF-FM and/or CellROX events.

We were further interested in examining the anti-microbial defenses exerted by recruited leukocytes at the injury site. Both ROS and NO represent critical defense mechanisms deployed by leukocytes against invading pathogens (46). While there was no increase in ROS levels at 12 hpi, a significantly higher percentage of leukocytes showed evidence of ROS activity at 24 and 48 hpi (**Figure 5**). This was followed by a sudden drop in ROS at 72 hpi (**Figure 5**). The upsurge in ROS production was associated with upregulation of pro-inflammatory cytokines (*TNFA* and*IL1B*) (**Figure 3**) along with the increase in neutrophil-centric leukocytic infiltration (**Figures 4A, B**) between 24 and 48 hpi. Additionally, a neutrophil-mediated decline in leukocyte numbers (**Figure 4**) as well as upregulation of *IL10* and *TGFB* gene expression (**Figure 3**), were correlated with a substantial drop in ROS at 72 hpi, suggesting a shift from pro-inflammatory to anti-inflammatory profile.

On the other hand, levels of another evolutionarily conserved defense mechanism, NO, were relatively lower than ROS at 24 and 48 hpi (**Figure 5**). NO is a signaling molecule that mediates anti-microbial activities (47–51), in addition to regulation of cellular and biological functions such as angiogenesis and chemotaxis (52–54). The percentage of leukocytes with NO activity increased gradually to reach a peak at 24 hpi. Although we detected a marked reduction in ROS at 72 hpi, levels of NO decreased gradually at 24 and 48 hpi (**Figure 5**). NO is synthesized by three different nitric oxide synthases (NOS): endothelial NOS (eNOS), neuronal NOS (nNOS) and inducible NOS (iNOS). Both eNOS and nNOS are constitutively expressed in endothelial cells and neurons, respectively. Meanwhile, iNOS expression is regulated in various cells, e.g., macrophages, monocytes, and mast cells, in response to inflammatory mediators (52, 55). Our data showed that *INOSA* gene expression was significantly upregulated at 24 hpi (**Figure 3**).

## 4 Discussion

The inflammatory response following skin injury is crucial for eradication of infection and normal wound healing (56). Following cutaneous injury, the innate immune system is activated *via* binding of DAMPs and PAMPs to PRRs expressed by tissue-resident cells (57). Induction of acute inflammatory response involves upregulation of pro-inflammatory cytokines and leukocyte migration to the infection site (5). Crucial to pathogen clearance and subsequent tissue repair is the recruitment of leukocytes from nearby blood vessels to the wound area (46, 58–60). Although our cumulative observations, in this study together with our previous published works (20), point to *A. veronii* playing a prominent role in induction of cutaneous immune responses, we cannot argue against additional potential contributions from deviations in microflora composition, opportunistic infections derived from this microbiota or incoming facility water, and/or the initial wounding procedure. Thus, we highlight all of these sources as potential contributors, and consider this model as representing the conditions often encountered in an aquatic facility infection where *A. veronii* is involved, rather than a controlled *A. veronii* infection. Hence, our results cannot be conclusively *A. veronii*-related yet they characterize the overall cutaneous inflammatory responses in goldfish.

Our data revealed a significant increase in leukocytes infiltrating the infection site at 36-48 hpi. Among infiltrating cells, neutrophils accounted for a significant portion when compared to monocyte/macrophage. Following infection, neutrophils are usually the first line of defense against infection since they infiltrate the infection site rapidly to become the dominant leukocyte in earlier stages of acute inflammation (56). In both mammals and fish, neutrophils exist in the bloodstream and to a larger extent within hematopoietic tissue, ready to migrate to circulation in response to a microbial challenge (61–64). Previously, our lab has shown a rapid mobilization of neutrophils from hematopoietic tissue to the circulation in response to *A. veronii* cutaneous infection (20). Though, the kinetics of neutrophil recruitment to the wound site were not established. In the present study, we observed neutrophil-centric leukocyte recruitment to the infection site, which is consistent with the period of neutrophilia detected at the first 48 hpi (20). Neutrophils were rarely detected at wound area at 0 hpi and increased significantly at 36-48 hpi, making neutrophils the main cells to respond and to be recruited to the infection site.

Neutrophil recruitment was associated with remarkable upregulation of pro-inflammatory cytokines (*TNFA* and *IL1B*) and a potent neutrophil chemoattractant (*CXCL8*). Pro-inflammatory cytokines were reported to enhance migration of immune cells, including neutrophils, to the infection site and further increase the level of chemoattractants to augment leukocyte recruitment (56). Downregulation of pro-inflammatory mediators and upregulation of anti-inflammatory cytokines (*TGFB* and *IL10*) were coupled to an abrupt decline in neutrophil numbers at 72 hpi, which led to a decline in total leukocytes infiltrating the wound area. Reduction in neutrophils at the infection site is possibly induced by their retrograde migration to the circulation (65) and cellular apoptosis (64). Our lab has previously shown a critical role of neutrophils in inflammation resolution *via* the formation of apoptotic bodies that are subsequently internalized by macrophages to initiate anti-inflammatory/pro-resolution programs (64).

Macrophages/monocytes were the main cells occupying the skin tissue (~75%) at 0 hpi, suggesting their role in immune surveillance, pathogen detection, induction of acute inflammatory response and recruitment of other leukocytes. We observed a relatively less increase in macrophage/monocyte numbers at 24-48 hpi when compared with neutrophils. This could be explained by the necessity of early neutrophil recruitment, thereby contributing to the majority of leukocytes infiltrating the wound area during the early phases of inflammation, which was largely at the expense of monocytes/macrophages. Interestingly, *A. veronii* cutaneous infection was shown to be associated with low levels of blood monocytes (20), in addition to their capacity to induce high levels of apoptosis in macrophage populations (17, 66), which may explain the relatively low numbers of monocytes/macrophages at the infection site. In contrast with *A. veronii*-induced neutrophilia, the substantially lower monocyte number in the peripheral circulation was attributed to an *A. veronii*-mediated selective recruitment of neutrophils (20), suggesting that the vascular route may not be the only path for monocyte recruitment to wound site. Recent studies indicated that monocyte migration to the infection site could be achieved through visceral organs (67). Moreover, a local proliferation of macrophages could contribute to the total monocyte/macrophage population detected at the wound area (68, 69).

Concomitantly with the significant drop in neutrophil numbers at 72 hpi, monocytes/macrophages and lymphocytes remained the dominant populations of leukocytes at the wound area (~ 40% and ~ 35%, respectively). Macrophages contribute significantly to the resolution of inflammation and subsequent tissue repair *via* upregulation of anti-inflammatory cytokines, heat shock proteins and growth factors (70). Activation of tissue machinery is critical for restoring tissue integrity and homeostasis following an injury. The process involves a cross-talk between several pathways and growth factors (71). Among these factors, the pleiotropic *VEGF* is considered crucial for several wound healing processes such as angiogenesis, reepithelialization and collagen deposition, in addition to enhancing vascular permeability to promote cellular chemotaxis (12, 44). qPCR analysis showed a significant increase in *VEGF* gene expression at 48, 72 and 96 hpi, suggesting a shift from inflammatory to proliferative phase in order to promote wound repair. Lymphocytes, on the other hand, infiltrated the wound area at 48-72 hpi to mediate antigen-specific responses that activate adaptive immune arm. Previous studies reported lymphocyte infiltration during the late inflammatory phase of wound healing to play a role in resolution of inflammation and tissue remodeling (72, 73).

We examined the activity of two evolutionally conserved defense mechanisms exerted by skin leukocytes, i.e., ROS and NO. Redox molecules, including NO and ROS as well as their products, e.g., hydrogen peroxide (H_2_O_2_), superoxide anion $\left(O_{2}^{-}\right)$ , and reactive nitrogenous species (RNS), are essential for regulating inflammatory responses and eradication of pathogens (74). ROS has a critical role in intracellular signaling pathways as well as anti-microbial activities (75). In response to inflammatory mediators and phagocytosis, ROS is generated by nicotinamide adenine dinucleotide phosphate-oxidase (NADPH oxidase) enzyme complex (76). At the inner wall of the phagosome, NADPH oxidase produces $O_{2}^{-}$ and H_2_O_2_ to destroy pathogens through damaging proteins, lipids and/or DNA (75, 77). Our data showed a remarkable increase in ROS levels associated with leukocytes isolated at 24 and 48 hpi followed by a sudden drop in ROS levels at 72 hpi. High ROS levels were coupled to a significant increase in the neutrophil-dominated leukocytes as well as an upsurge in the gene expression of pro-inflammatory cytokines. Likewise, a reduction in neutrophil numbers correlated with a marked decline in ROS at 72 hpi.

NO level, in contrast, was relatively lower than ROS. After reaching a peak at 24 hours, its level did not change dramatically when compared to ROS. This could be attributed to the constitutive basal expression level of NO by both eNOS and nNOS as well as its pivotal role in other biological processes beyond immunological functions. NO possesses anti-microbial properties, including suppression of bacterial DNA repair and enzymes (47–51). Furthermore, NO enhances respiratory burst-induced cytotoxicity in bacterial cells (78) and protects against oxidative stress-associated cellular injury (79) *via* controlling ROS production and minimizing the reactivity of $O_{2}^{-}$ and H_2_O_2_ (74). Recently, our lab showed a reverse relationship between the levels of NO and ROS (data not published), where high levels of NO were coupled to low ROS in behavioural fever fish model. This may justify our findings where a relatively low NO production was detected along with high ROS levels at the same time points.

Resolution of cutaneous inflammation was detected at 72 hpi, indicated by a neutrophil-driven reduction in leukocytes, downregulation of pro-inflammatory mediators and decreased ROS. These pro-resolution events were potentially driven by anti-inflammatory cytokines, e.g., *IL10* and *TGFB* that were upregulated at 72 and 96 hpi. *IL10* was found to downregulate NADPH oxidase essential for ROS generation (39). Meanwhile, *TGFB* reduces levels of pro-inflammatory cytokines that potentiate leukocyte recruitment and ROS production (38, 40). Even though it is crucial for pathogen killing and intracellular signaling, ROS may injure the host tissue if released extracellularly in large quantities, resulting in chronic inflammation and impaired wound healing (80). Therefore, the balance between pro-inflammatory and anti-inflammatory mediators is crucial for effective pathogen clearance along with minimal collateral tissue damage (6). Resolution of inflammation is suggested to be mediated by several mechanisms. For instance, macrophages internalize apoptotic neutrophils followed by downregulation of their pro-inflammatory profile (9–11). Additionally, activated neutrophils *ex vivo* also showed the capacity to engulf apoptotic cells, leading to inflammation control and a substantial reduction in ROS (11, 81).

**Figure 6:**
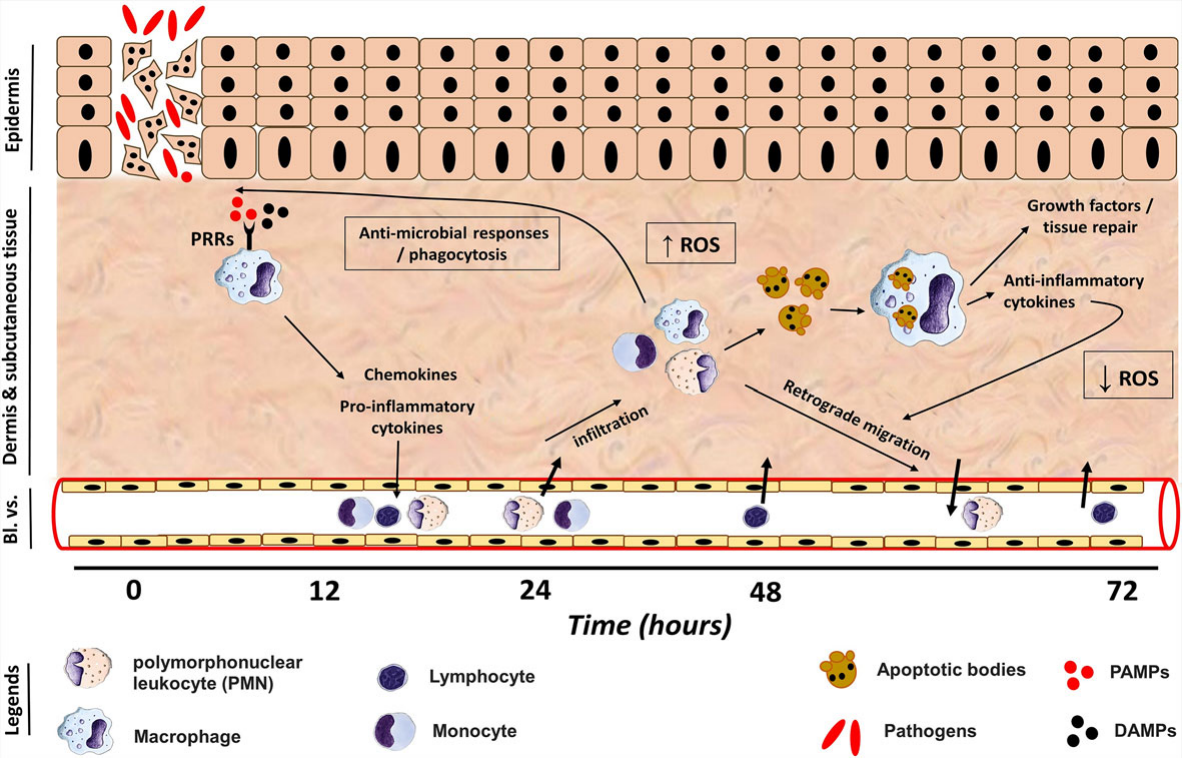
Phagocyte immune response during induction and resolution phases of cutaneous inflammation. Damage/Pathogen Associated Molecular Patterns (DAMPs/PAMPs) induced by an injury and/or infection bind to pattern recognition receptors (PRRs) expressed by tissue resident macrophages, which in turn release pro-inflammatory cytokines and chemokines to trigger an acute inflammatory response and to recruit leukocytes to the injury site from nearby blood vessels (Bl. *vs*.). Polymorphonuclear (PMN) leukocytes infiltrate the wound site gradually to reach a peak at 36-48 hpi, where they and macrophages exert antimicrobial defense mechanism including reactive oxygen species (ROS), to combat pathogens. The peak of ROS was at 24-48 hpi, which was coupled to an increase in PMN number at the wound site. (Lym) lymphocytes were noticed to infiltrate the injury site at 48 and 72 hpi. Following elimination of pathogens, PMN undergo cellular apoptosis to release chemoattractant to attract macrophages, which engulf apoptotic PMN. Activated macrophages release anti-inflammatory cytokines to provoke the resolution of inflammation *via* suppression of ROS production and a retrograde migration of PMN back to circulation at 72 hpi. Furthermore, macrophages release growth factors to activate tissue repair machinery and restore homeostasis.

Collectively, our data suggest that subsequent to cutaneous injury infected with *A. veronii*, an acute inflammatory response peaked at 24-48 hpi, identified by a neutrophil-dominated migration of leukocytes to the infection site, where they deployed anti-microbial defenses (i.e., ROS and NO) to combat pathogens (**Figure 6**). The acute inflammatory response and leukocyte recruitment are likely triggered and regulated *via* pro-inflammatory cytokines such as *TNFA* and *IL1B* as well as chemokines including *CXCL8* (4, 5) (**Figure 6**). A shift from pro-inflammatory to pro-resolution state was noticed at 72 hpi, evident by a substantial drop in neutrophils, which was possibly induced by a retrograde migration of PMN back into the circulation (65) and neutrophil apoptosis (64) (**Figure 6**). PMN undergo apoptosis and produce chemotactic factors to attract macrophages (65, 82), which in turn engulf apoptotic bodies and secrete pro-resolution/anti-inflammatory cytokines, e.g., *TGFB* and *IL10* as well as growth factors (83). Anti-inflammatory cytokines provoke a reduction in the pro-inflammatory mediators and ROS production to control inflammation (40) (**Figure 6**). Meanwhile, growth factors are critical for the activation of tissue repair machinery to restore integrity and homeostasis. Lastly, the reported model system can be utilized for studying skin diseases and a wide variety of other biological processes, including basic immunology, evolutionary and developmental biology (84–86).

## Data Availability Statement

The raw data of this article will be made available by the authors without reservation.

## Ethics Statement

The animal study was reviewed and approved by as per guidelines specified by the Canadian Council on Animal Care, and protocols approved by the University of Alberta Animal Care and Use Committee (protocol AUP0000706).

## Author Contributions

AS conceived and designed the experiments, performed the experiments, analyzed the data, and wrote the manuscript. TY and JW conceived and designed the experiments, performed the experiments. JS and DB conceived and designed the experiments, analyzed the data, and wrote the manuscript. All authors contributed to the article and approved the submitted version.

## Funding

This work was supported by the Natural Sciences and Engineering Council of Canada grant to DRB (RGPIN-2018-05768). 

## Conflict of Interest

The authors declare that the research was conducted in the absence of any commercial or financial relationships that could be construed as a potential conflict of interest.

## Publisher’s Note

All claims expressed in this article are solely those of the authors and do not necessarily represent those of their affiliated organizations, or those of the publisher, the editors and the reviewers. Any product that may be evaluated in this article, or claim that may be made by its manufacturer, is not guaranteed or endorsed by the publisher.

## Supplementary Material

Supplementary Figure 1 Gating strategy to evaluate the production of reactive oxygen species (ROS) and nitric oxide (NO). **(A)** Cells acquired in an ImageStream Mk II Imaging Flow Cytometer (Amnis) were gated using gradient RMS to identify focused cells. Single cells **(B)** were subsequently evaluated for viability based on propidium iodide staining **(C)**. ROS and NO were examined based on CellROX **(D)** and DAF-FM **(E)** staining, respectively. BF, bright field.
