# Supplementary figures and images for: Isolation of Skin Leukocytes Uncovers Phagocyte Inflammatory Responses During Induction and Resolution of Cutaneous Inflammation in Fish
Source: Front Immunol. 2021 Sep 24;12:725063. doi: 10.3389/fimmu.2021.725063 (PMC8497900; doi:10.3389/fimmu.2021.725063)

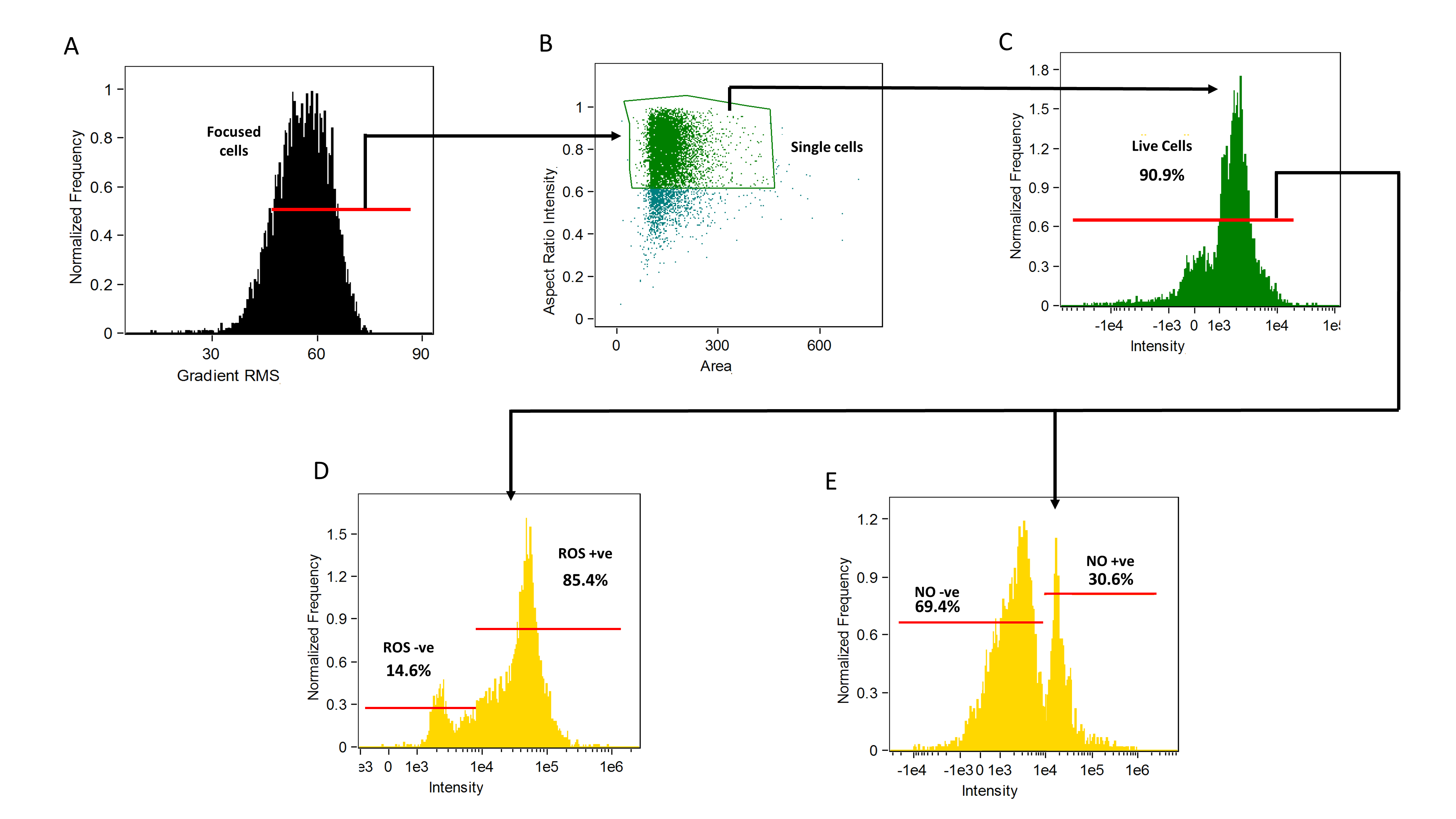

Supplement: Supplementary Figure 1 — Gating strategy to evaluate the production of reactive oxygen species (ROS) and nitric oxide (NO). (A) Cells acquired in an ImageStream Mk II Imaging Flow Cytometer (Amnis) were gated using gradient RMS to identify focused cells. Single cells (B) were subsequently evaluated for viability based on propidium iodide staining (C). ROS and NO were examined based on CellROX (D) and DAF-FM (E) staining, respectively. BF, bright field. [file Image_1.tif]
